# A new surgical technique for short bowel syndrome

**DOI:** 10.1186/s12893-022-01823-5

**Published:** 2022-11-03

**Authors:** Isamu Saeki, Sho Kurihara, Masato Kojima, Hiroki Ohge, Shinya Takahashi, Eiso Hiyama

**Affiliations:** 1 Department of Pediatric Surgery, Hiroshima University Hospital, 1-2-3 Kasumi Minami-Ku, Hiroshima-Shi, Hiroshima 734-8551 Japan; 2 Department of Surgery, Hiroshima University Hospital, Hiroshima, Japan; 3 Department of Cardiovascular Surgery, Hiroshima University Hospital, Hiroshima, Japan

**Keywords:** Short bowel syndrome, Small intestine, Intestinal rehabilitation, Biomimetics, Surgical technique

## Abstract

**Objective:**

Short bowel syndrome (SBS) is a severe intestinal disease that causes malabsorption. Long-term parental nutrition therapy induces infection and liver failure. For the surgical management of intestinal rehabilitation, the intestinal loop lengthening method and serial transverse enteroplasty (STEP) method have been reported, although their effects have proven limited. We herein report a new surgical technique, Saeki–Spiral–Shark (3S) method for SBS using biomimetics of shark intestine.

**Methods:**

In the 3S method, a spiral valve is formed inside the intestine by external sutures. Using a 25 cm length intestinal organ model, we performed both the 3S method and STEP procedure. We then compared the length and fluid passage times of the subsequently formed intestine.

**Results:**

After the 3S method was performed, the length of the intestinal model changed to 22 cm, and after the STEP procedure, that was elongated to 30 cm. Although the water passage times did not change markedly, the semi-digestive nutritional supplement passage time slowed down in the model with the 3S method. There was slight leakage in the STEP procedure model.

**Conclusions:**

The 3S method is a unique method of treating SBS based on biomimetics. This procedure does not require an incision of the intestine, which thereby enabling clean and less-invasive surgery. We plan to conduct animal experiments in the future.

## Introduction

Short bowel syndrome (SBS) is defined as a congenital or acquired massive reduction in the length of the small intestine. Various diseases (e.g. midgut volvulus, necrotizing enterocolitis, gastroschisis, strangulation ileus, mesenteric ischemia, inflammatory bowel disease) cause SBS [1]. SBS patients require parental nutrition (PN), which sometimes causes significant complications, such as sepsis or intestinal failure-associated liver disease [2, 3]. Surgical management is sometimes selected for intestinal rehabilitation. As an intestinal lengthening technique, Bianchi [4] reported an intestinal loop lengthening method, and Kim et al. [5] reported serial transverse enteroplasty (STEP). In some reports, the STEP procedure is said to aid intestinal adaptation and improve the long-term outcome [6, 7]. However, the treatment results are insufficient at present. A new breakthrough in surgical treatments for SBS is thus desired.

**Fig. 1:**
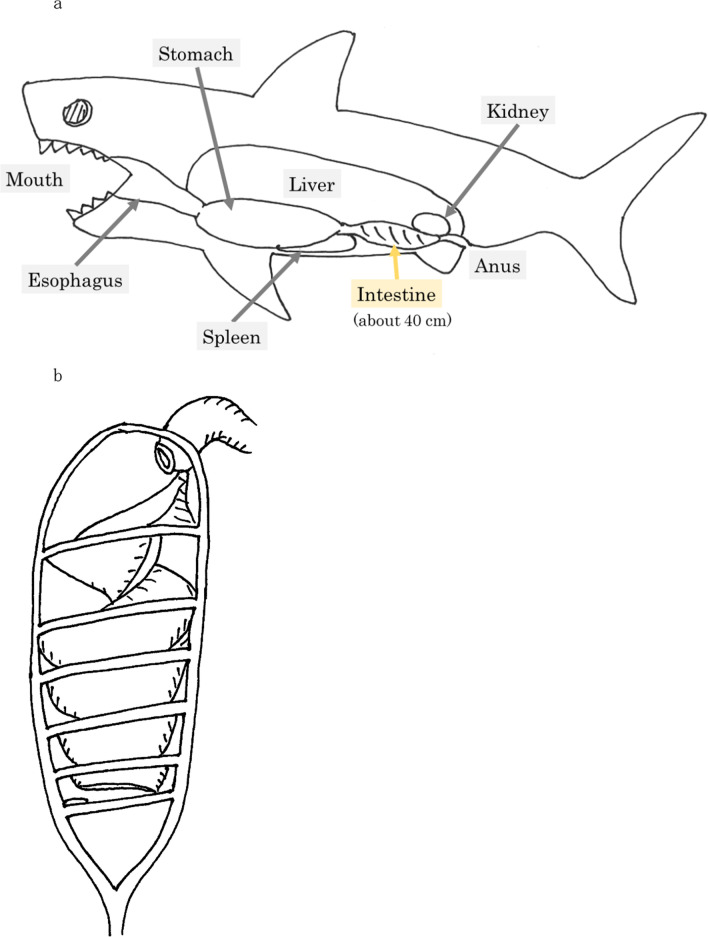
Spiral valves in the shark intestine. **a** Sharks have a very short intestine. **b** Sharks’ intestine has a unique structure. The contents flow slowly along the spiral valves

Technology that incorporates useful characteristics of other living beings is referred to as biomimetics [8]. Sharks have a unique intestinal structure. Despite a very short intestine (about 40 cm in length), they swallow and digest fish without problems (Fig. 1a). Some of them have spiral valves in their intestine, and the contents flow slowly along them (Fig. 1b). Leigh et al. [9] suggested that shark spiral intestines may operate as Tesla valves, which may aid in effective absorption.

We herein report a newly devised surgical technique for SBS using biomimetics derived from shark intestine.

### Methods

In vitro fluid passage experiments were performed using a surgical intestinal model (WetLab Ohtsu, Japan). The intestinal models were 25 cm in length and 4 cm in diameter.

### 3S method (Fig. 2a–c)

**Fig. 2:**
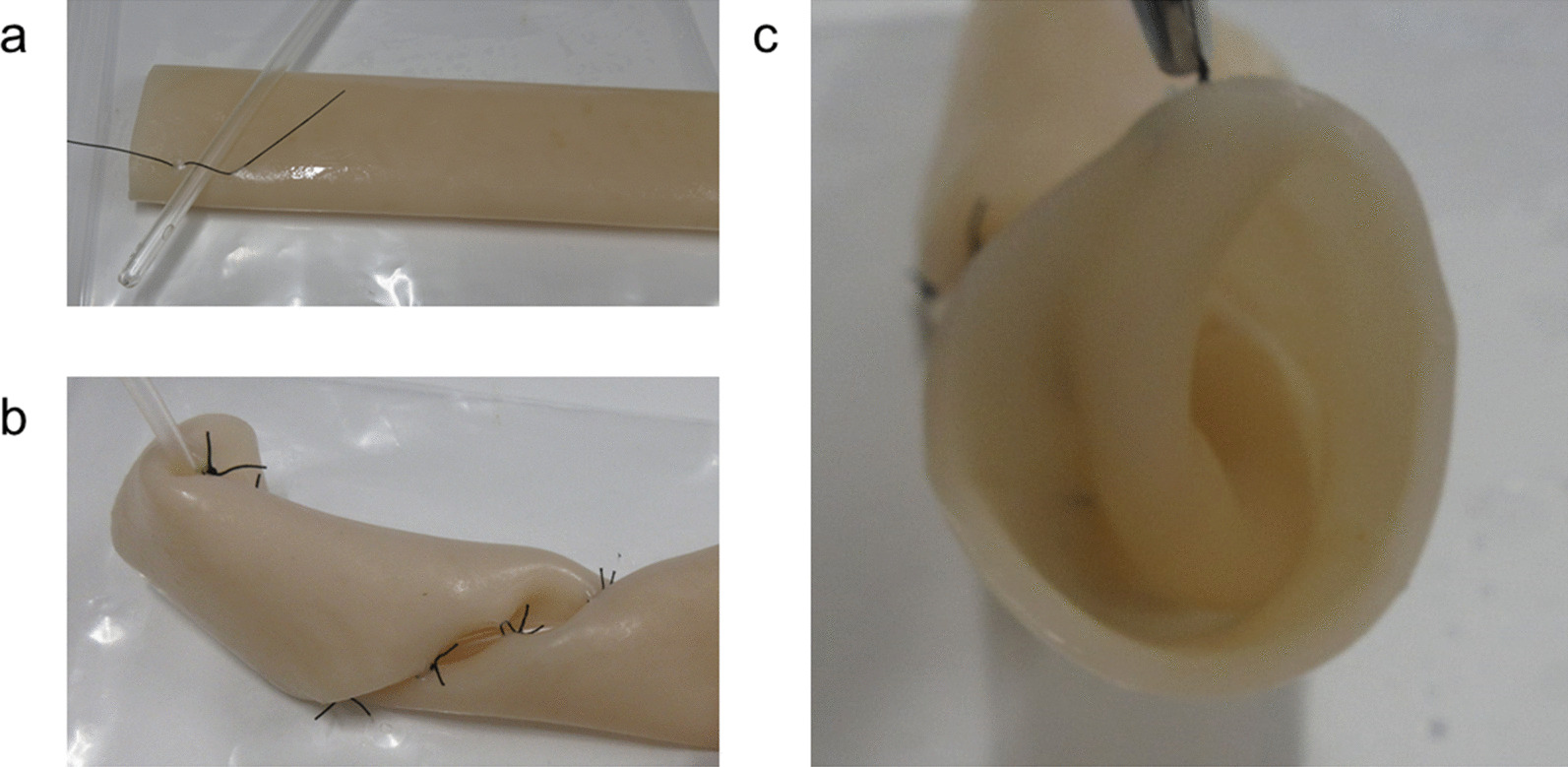
3S method. a A soft so-called Nelaton catheter is attached to the intestine at about 45° as a guide. b Nodule sutures are placed in the intestine along the catheter to create a spiral intestinal valve inside the intestine. c With the 3S method, a spiral valve is formed inside the intestine

A soft so-called Nelaton catheter was attached to the intestine at about 45° as a guide (Fig. 2a). Nodule sutures were placed in the intestine along the catheter to create a spiral intestinal valve inside the intestine (Fig. 2b). The Nelaton catheter was then removed, leaving the spiral valve inside the intestine (Fig. 2c). We named this procedure as the Saeki–Spiral–Shark (3S) method.

### STEP procedure

Incisions of 2 cm and continuous sutures were performed alternately in the 90° and 270° positions at 7 places with 3 cm intervals. Kim et al. [5] performed long, dence incisions of an animal model intestine using a GIA stapler in their initial reports. Although we first perfromed 2.5 cm incisions and placed continuous sutures alternately 90° and 270° positions at 2 cm intervals, the passage was extremely prohibited with massive leakage. We therefore selected the above incision size and interval instead.

### Fluid passage experiments

**Fig. 3:**
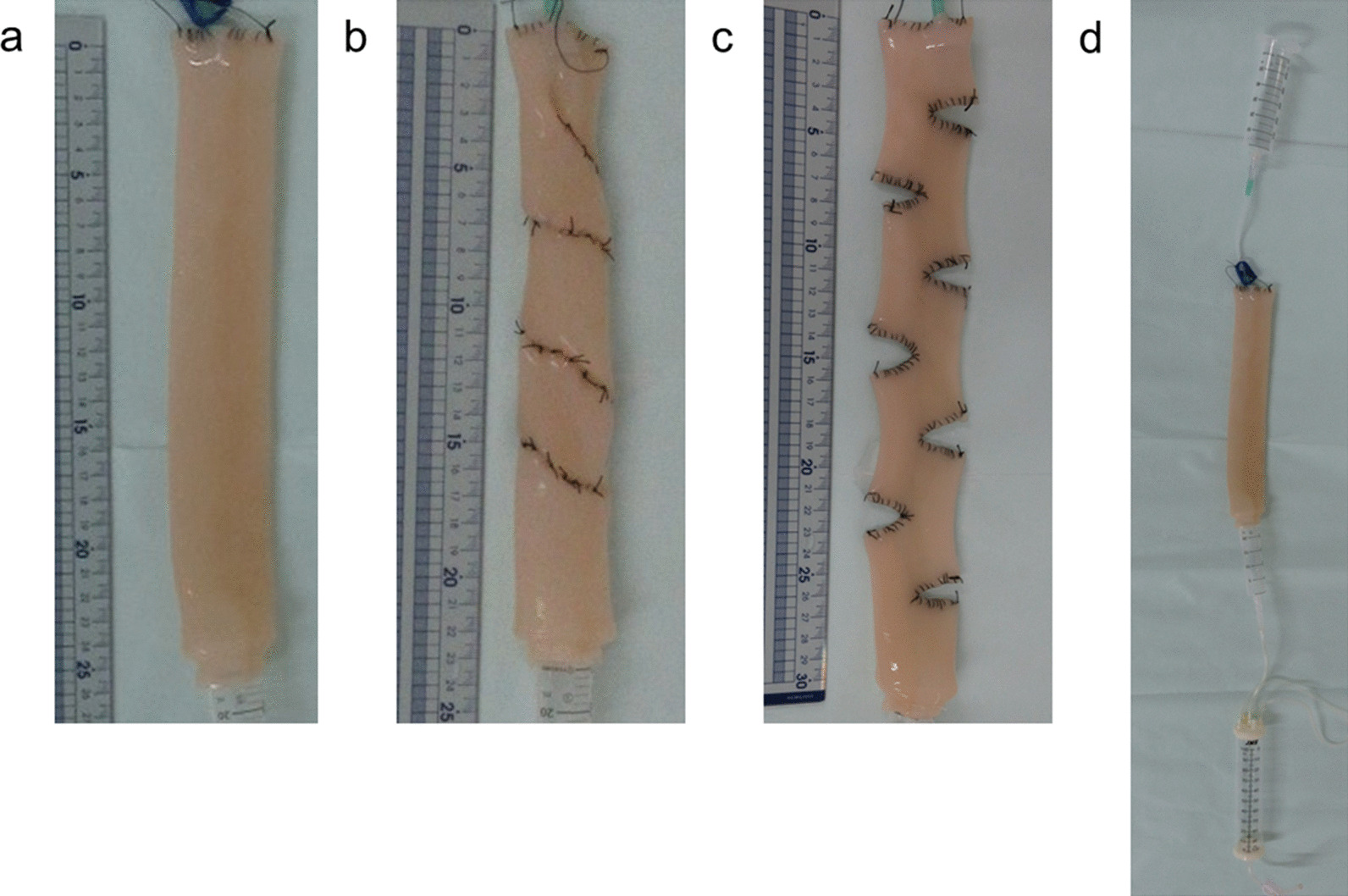
In vitro passage experiment. **a** Straight intestine (without surgery), 25 cm in length. **b** 3S method, 22 cm in length. **c** STEP procedure, 30 cm in length. **d** A total of 50 ml of fluid dripped freely from the upper side of the intestine, and the passage amount was measured every 15 s

We performed fluid passage experiments using three types of intestine (straight: with no procedure, 3S method, and STEP procedure). The length of straight intestine was 25 cm (Fig. 3a). After the 3S method, the length changed to 22 cm, and after the STEP procedure, the length changed to 30 cm (Fig. 3b, c). The outer diameter at the narrowest part of the experimental intestine had changed from 4 to 2 cm after the STEP procedure and 3.2 cm after the 3S method. At the narrowest part, the inside diameter changed from 3.8 cm to 1.8 cm after the STEP procedure and 2.4 cm after the 3S method in. A total of 50 ml of fluid (water and semi-digestive nutritional supplement with a viscosity of 17 mPa·s) was dripped freely from the upper side of the intestine, and the passage amount was measured every 15 s (Fig. 3d). Measurement were repeated five times each and the average result was determined.

## Results

**Fig. 4:**
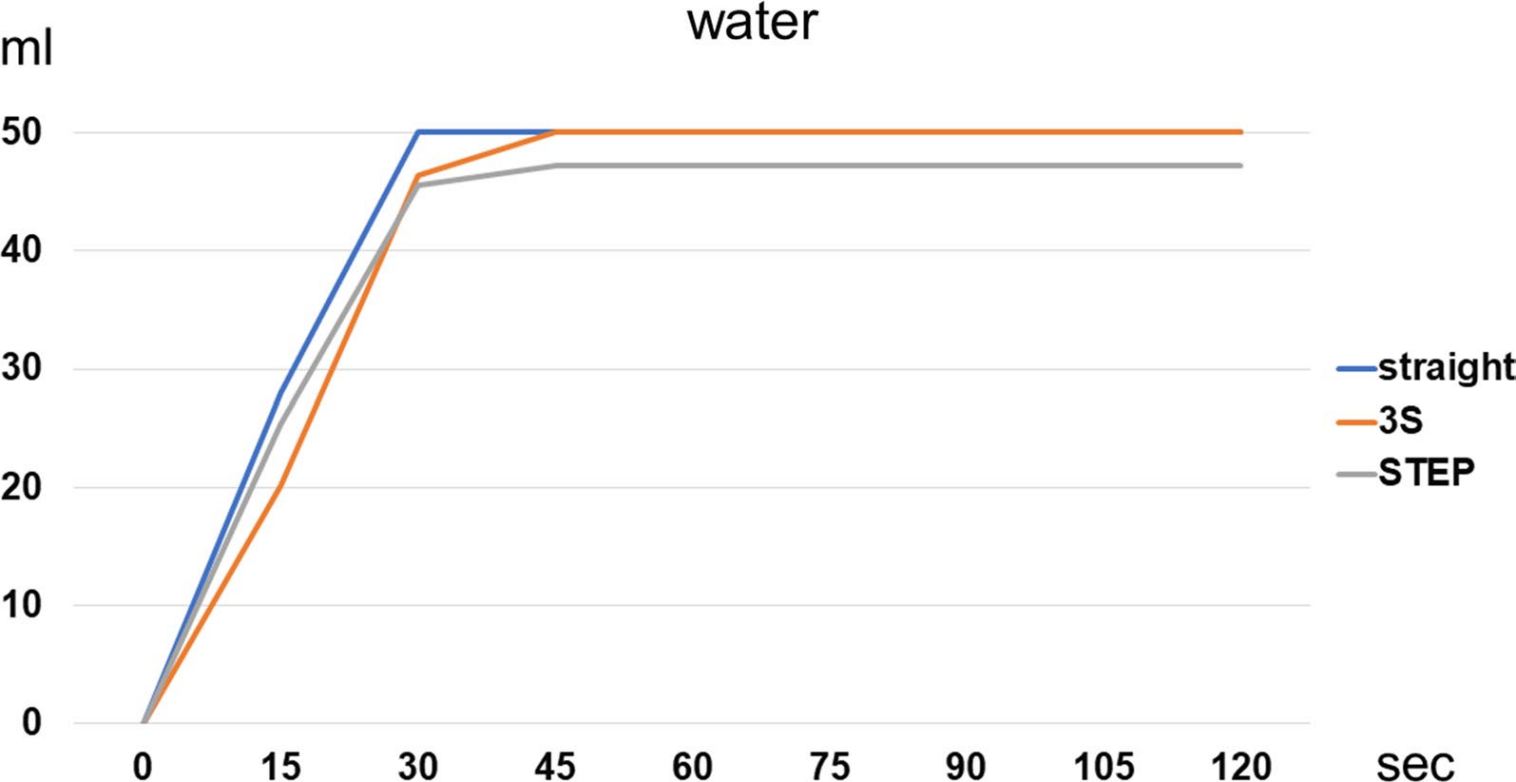
Water passage time. Water rapidly passed through the intestines, showing almost no differences among the three types of intestine. The total amount of water passage with the STEP procedure did not reach the full amount (50 ml) because of leakage

Figure 4 shows the water passage times (Fig. 4). Water rapidly passed through these intestine models, without remarkable differences noted in the three type models of intestine. The total amount of water passage following in the STEP procedure model did not reach the full amount (50 ml) because of leakage.

**Fig. 5:**
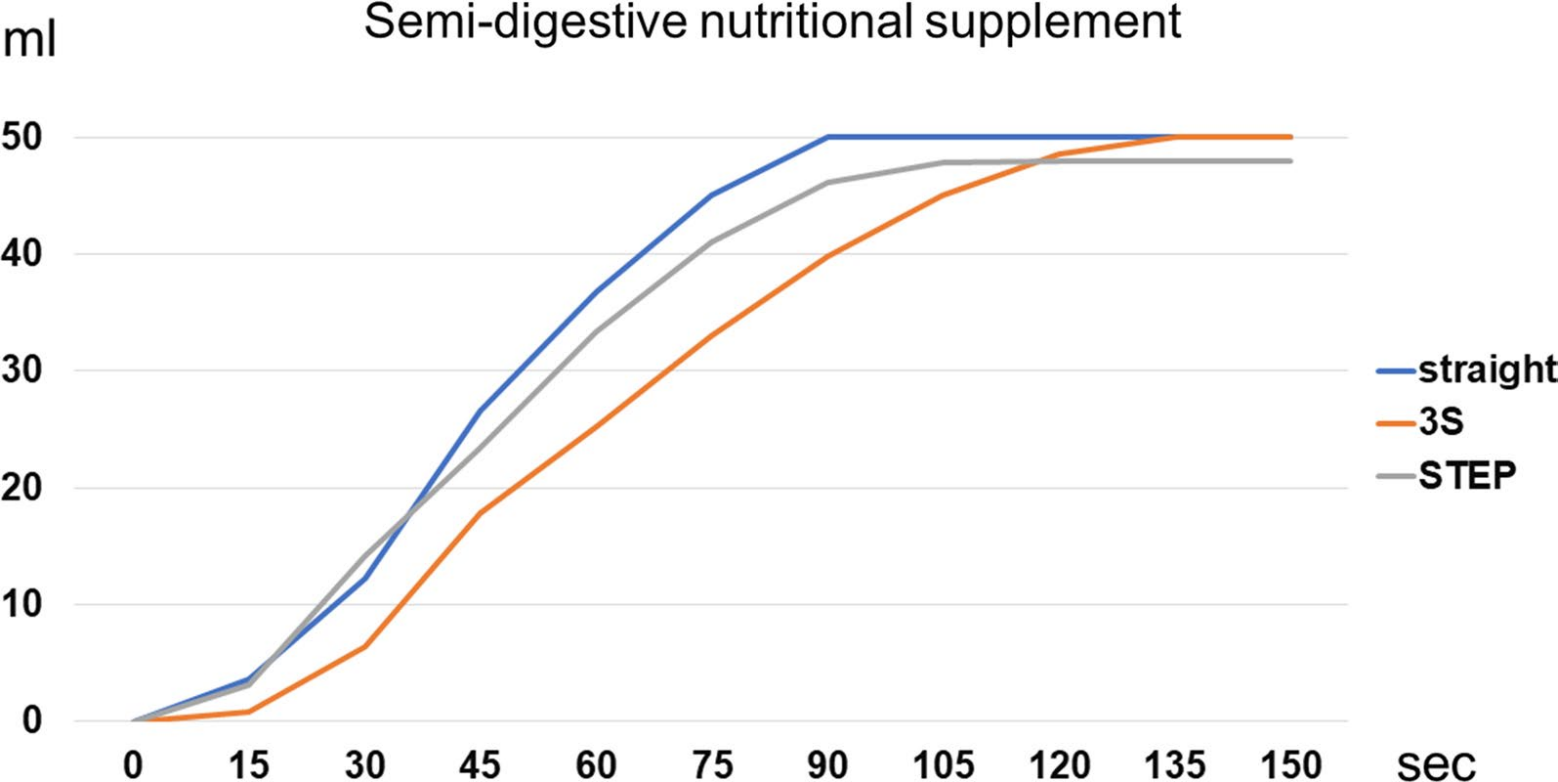
Semi-digestive nutritional supplement passage time. The semi-digestive nutritional supplement, which has higher viscosity than water, slowly passed through the intestine with the 3S method. The total amount of supplement passage with the STEP procedure did not reach the full amount (50 ml) because of leakage

Figure 5 shows the semi-digestive nutritional supplement passage times (Fig. 5). The semi-digestive nutritional supplement, which has a higher viscosity than water, most slowly passed through the intestine following the 3S method. The total amount of supplement passage in the 3S model is the same of the loaded volume but the STEP model did not reach the full amount (50 ml) because of leakage.

## Discussion

SBS is a very serious condition, and both medical treatment as well as various surgical approaches have been reported for its management, including artificial intestinal valve, impaired propulsive peristalsis, tapering of dilated intestine, intestinal loop lengthening and STEP [4, 5, 10–13]. A final message of satisfactory surgical treatment for SBS is consequently intestinal transplantation [3, 14]. The current main-stream surgical approach is STEP, and with it, a number of patients can reduce their need for or withdraw entirely from PN [6, 7]. However, STEP and many other methods require many incisions and sutures to the intestine, which raise serious intestinal damage and sometimes cause infections or leakage after surgery.

The 3S method is a unique procedure in which many folds are formed inside the intestine by externally placed sutures. Tapering of the dilated intestine and placement of artificial intestinal valves are performed simultaneously. In addition, this method does not decrease the intestinal surface area for absorption by sutures. Although the 3S method is admittedly similar to the artificial intestinal valve method in terms of forming valves inside the intestine [10], the novel point with the 3S approach is “spiral intestinal valve formation”, which we adapted from shark intestine via biomimetics. Although the 3S method appears simple at first glance, this spiral valve structure is based on fluid mechanics and is extremely reasonable.

Most surgeries for SBS focus on lengthening the bowel. STEP, Bianchi (intestinal loop lengthening), and the spiral intestinal lengthening and tailoring (SILT) procedure all have an intestinal lengthening effect [4–6, 11, 12]. SILT provides a spiral configuration to the bowel and at the same time provides lengthening, which is similar to our 3S method in the point of using a spiral [11, 12]. The 3S method is not an intestinal lengthening procedure, rather it is an intestinal valve formation method. Although the 3S method makes the intestine shorter, the Tesla valve effect may help to slow the contents. Although the total length of the intestine becomes shorter after the 3S method, the flow speed of the contents in the intestine becomse slower. In the present study, our fundamental experiments using a model intestinal organ showed that the 3S method significantly slowed the passage of a high-viscosity fluid (semi-digestive nutritional supplement) through the intestine, which may help increase the absorption efficiency.

Another break through aspect of the 3S method is unrequirement of intestinal incision. In our experiments, while there was no leakage with the 3S method at all, slight fluid leakage occurred in the STEP procedure. In common clinical surgery, a GIA stapler is used to perform the STEP procedure, which helps reduce but not entirely eliminate the risk of leakage. The 3S method does not require any intestinal incisions or anastomosis, so the surgical cleanliness and safety are highly retained.

The 3S method is an intestinal valve formation method that narrows the intestine by folding the intestinal wall. We recommend that the 3S method only be performed for the dilated intestine.

As a limitation, the real comparison of the 3S method and the STEP procedure was inadequate due to the presence of leakage. Almost all the leakage occurs at the needle hole of the first cut and the suture point of the STEP procedure. In the STEP procedure, the inside diameter of the intestine narrows abruptly from 3.8 cm to 1.8 cm. This may cause a small amount of leakage. We used experimental intestine in our study. In an actual animal experiment, this small amount of leakage will disappear because of the healing ability. Further study is needed to perform a more precise evaluation.

This study was a fundamental experiment using an intestinal organ model. We plan to conduct animal experiments in the future to determine whether or not our 3S method actually improves intestinal absorption in SBS.

## Data Availability

The datasets used and analyzed during the current study available from the corresponding author on reasonable request.

## Abbreviations

SBS: Short bowel syndrome
PN: Parental nutrition
STEP: Serial transverse enteroplasty
SILT: Spiral intestinal lengthening and tailoring
